# A public health intervention package for increasing tuberculosis notifications from private practitioners in Bandung, Indonesia (INSTEP2): A cluster-randomised controlled trial protocol

**DOI:** 10.12688/f1000research.52089.2

**Published:** 2022-03-25

**Authors:** Panji Fortuna Hadisoemarto, Bony Wiem Lestari, Katrina Sharples, Nur Afifah, Lidya Chaidir, Chuan-Chin Huang, Susan McAllister, Reinout van Crevel, Megan Murray, Bachti Alisjahbana, Philip C Hill

**Affiliations:** 1 Tuberculosis Working Group, Infectious Disease Research Centre, Faculty of Medicine Universitas Padjadjaran, Bandung, West Java, 40161, Indonesia; 2 Department of Public Health, Faculty of Medicine Universitas Padjadjaran, Bandung, West Java, 40161, Indonesia; 3 Department of Preventive and Social Medicine, Centre for International Health, University of Otago, Dunedin, 9016, New Zealand; 4 Department of Internal Medicine, Radboud Institute for Health Sciences, Radboud University Medical Centre, Nijmegen, 6525 GA, The Netherlands; 5 Department of Mathematics and Statistics, University of Otago, Dunedin, 9016, New Zealand; 6 Department of Microbiology, Faculty of Medicine Universitas Padjadjaran, Bandung, West Java, 40161, Indonesia; 7 Division of Global Health Equity, Brigham and Women's Hospital, Harvard Medical School, Boston, Massachusetts, 02115, USA; 8 Department of Internal Medicine, Dr Hasan Sadikin General Hospital, Bandung, West Java, 40161, Indonesia

**Keywords:** tuberculosis, private practitioner, notification, protocol

## Abstract

**Background. **A significant proportion of tuberculosis (TB) patients globally make their initial visit for medical care to either an informal provider or a private practitioner, and many are not formally notified. Involvement of private practitioners (PPs) in a public–private mix for TB (TB-PPM) provides an opportunity for improving TB control. However, context-specific interventions beyond public–private agreements and mandatory notification are needed. In this study we will evaluate whether a tailored intervention package can increase TB notifications from PPs in Indonesia.

**Methods. **This is a cluster-randomized trial of a multi-component public health intervention. 36 community health centre (CHC) areas will be selected as study locations and randomly allocated to intervention and control arms (1:1). PPs in the intervention areas will be identified using a mapping exercise and recruited into the study if they are eligible and consent. They will receive a tailored intervention package including in-person education about TB management along with bimonthly electronic refreshers, context-specific selection of referral pathways, and access to a TB-reporting app developed in collaboration with the National TB programme. The primary hypothesis is that the intervention package will increase the TB notification rate. The primary outcome will be measured by collecting notification data from the CHCs in intervention and control arms at the end of a 1-year observation period and comparing with the 1-year pre-intervention. The primary analysis will be intention-to-treat at the cluster level, using a generalised mixed model with repeated measures of TB notifications for 1 year pre- and 1 year post-intervention.

**Discussion. **The results from this study will provide evidence on whether a tailored intervention package is effective in increasing the number of TB notifications, and whether the PPs refer presumptive TB cases correctly. The study results will guide policy in the development of TB-PPM in Indonesia and similar settings.

## Introduction

Tuberculosis (TB) is the leading infectious disease cause of global mortality. In 2018, an estimated 10 million people had TB and, including those living with HIV, 1.4 million died from the illness. However, approximately 3 million new TB cases are estimated to not be notified to health authorities, 2 million of which reside in the 20 high-burden countries that contribute 70% of the total global TB incidence. ^1^ Case notification plays an important role in disease surveillance by identifying cases as potential sources of new infections and their contacts, measuring the disease burden, and prompting the implementation of timely curative treatment. Achieving the End TB target of TB elimination by the year 2030 relies on the completeness of case notifications.

In Asian countries with high TB incidence, between 70–85% of TB patients first seek care in the private sector. ^2^ Among the different types of providers in the private sector, medically trained general practitioners or specialists who work either in solo practice or private clinics (private practitioners (PPs) play an important role because of their proximity to the community. An analysis of TB patient pathways in five low-middle income countries (LMICs) demonstrated that, on average, 26% of TB patients first sought care at primary care clinics, of whom only 13% of patients were tested with appropriate TB diagnostics, and only 7% were notified to the National TB Programme (NTP). ^3^ PPs’ knowledge about correct TB management is highly variable and provision of substandard TB treatment is not uncommon. ^4^ Hence, involving PPs in a TB public–private mix (TB-PPM) strategy has been recognized as an opportunity to improve TB control. It is challenging for NTPs to address these issues due to their scale, relatively unregulated PP practice, and limited resources. ^5^

There were an estimated 845,000 incident cases of TB in Indonesia in 2018, the third highest after India and China. ^1^ Approximately 20% of these are seen and treated in private, primary care health facilities. ^6^ However, according to Indonesia’s TB inventory study, 41% of the incident TB cases were not notified to the NTP. Among health facilities, PPs had the lowest reporting rate (4%) followed by public/private hospitals (38%), both of which were lower than publicly funded Community Health Centres (CHC) ( *Pusat Kesehatan Masyarakat/Puskesmas*) (85%). ^7^

Although TB-PPM has been recognized as an important component in Indonesia’s TB control programme, engagement of the private sector by donor-funded district-based TB-PPM pilot projects has been mostly limited to hospitals and specialists. ^2^ As a part of advancing TB-PPM, the government made TB notification mandatory in 2016, but evidence of impact and compliance is lacking. ^8^ In general, the benefits of PPM initiatives have yet to be fully realized across the country. ^9^

Interventions aimed at increasing PPs engagement need to be based on a sound understanding of contextual factors relevant to PPs and how PPs relate to the public sector with respect to the diagnosis, treatment and reporting of TB cases. ^10^ We have previously conducted a study to describe and understand healthcare pathways of patients seeking treatment for TB and the quality of TB case management by PPs in the city of Bandung. ^11^ In 30 CHC catchment areas, we identified 1200 PPs, of whom 245 had diagnosed at least one TB case in the previous 3 months. Among the 870 TB cases diagnosed by these PPs, fewer than 20% were notified. In an earlier feasibility study, ^12^ we showed that 40% of participating PPs had inadequate knowledge of TB symptoms and/or signs. A digital application designed to assist with referral and notification was well received, utilized and showed potential to increase TB notifications, although not all PPs had phones that supported it. In this cluster-randomised trial, we will evaluate a further-refined tailored intervention package to increase notifications of TB by PPs in Bandung, Indonesia.

### Objectives

We will evaluate, first, whether a tailored intervention package increases notifications of TB from PPs in Bandung, Indonesia by calculating the difference in the change in number of TB notifications after a 1-year intervention between intervention and control arms, and second, to measure the proportion of referrals from PPs to CHCs in the study arms that are actually diagnosed with TB.

## Methods

### Trial design

This study has been registered on clinicaltrials.gov, December 2019, NCT04187313. It is a cluster-randomised controlled trial of a multi-component public health intervention to increase notifications of TB from PPs in Bandung, in Indonesia. Clusters will be defined as CHC catchment areas and the intervention will be administered directly to PPs in areas randomised to the intervention arm. No intervention will be given to PPs in the control arm. The CHCs in both intervention and control arms will be informed about the study and asked, through the local Health Office, to make their notification data available. Notifications will be obtained directly from local NTP registry data, with accompanying information gathered about the address of the patient and referring doctor. Notified TB cases are, by definition, TB cases who have started TB treatment. Data on address of the patient will identify the extent of the “contamination” between arms.

### Study setting

**Figure 1.**
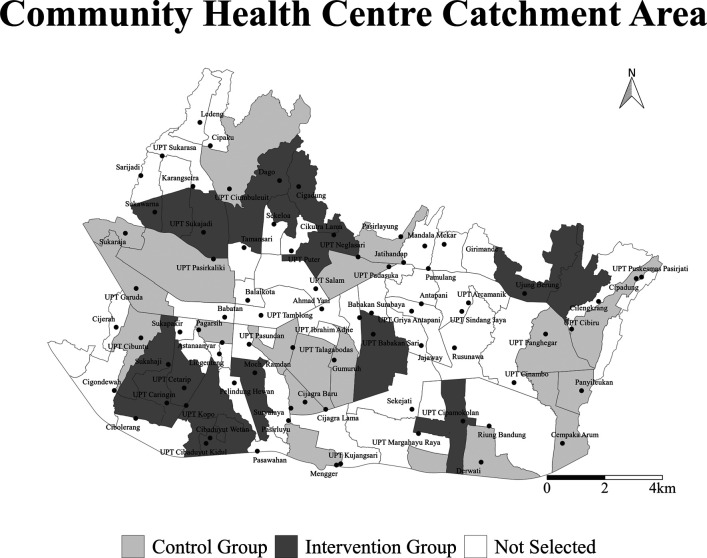
The map of intervention areas in Bandung. Thirty Community Health Centre (CHC) areas were randomly selected and assigned to the invention arm (dark gray shade) and control arm (light gray shade) for INSTEP2 study.

Bandung, a city of 2.5 million inhabitants, is served by 73 CHCs, each covering a defined geographic administrative area. Of these, 30 were randomly selected in proportion to population size for a previously conducted study. ^11^ The 30 selected CHCs will be randomly allocated into intervention and control arms with 15 CHCs for each arm ( Figure 1). The study population consists of all the people who may visit a PP in any of the selected CHC study areas during the year before and the year after the intervention. The outcome event is TB notification reported to the CHC by PPs in the selected study area.

### Eligibility criteria

All PPs in the intervention arm who reported having diagnosed at least one TB case in the past 3 months or who are practicing at the same private clinics where at least one PP reported having diagnosed at least one TB case in the past 3 months will be eligible for the intervention. PPs will be identified through a community-based mapping exercise conducted in our previous study and updated for the current study. Eligible PPs will intend to work in the current location for the duration of the study as their primary place of private practice. They will be ineligible if they are unable to use an electronic device for referral, anticipate more than 3 months of non-practice during the study period, or they are not qualified to practice according to the Indonesian Medical Association.

We will personally approach, at their place of work, each of the identified PPs in the intervention areas. They will be fully informed about the study, invited to participate, and provide written informed consent. A schedule will be generated for their education and their follow-up (once every two months) during the intervention period. We expect around 80% of approached PPs will be willing to participate in the study. PPs participating in the intervention will be offered a non-financial incentive of a certificate of proficiency issued by the Indonesian Medical Association that provides them with credits that can be used as proof of continuing medical education for renewing their license to practice.

### Intervention

The study intervention will comprise of three components:

**Intervention 1: Education.** We will develop educational material on TB management specifically aimed for PPs. According to the Indonesian National Standards for Medical Profession Education (2019), general practitioners ought to have been trained to identify, manage, and report uncomplicated pulmonary TB cases (level 4 competence), and to identify and refer suspected MDR-TB patients (level 3A competence). ^12^ Whereas internists and pulmonologists ought to have been trained to manage MDR-TB patients. However, in a survey of 547 general practitioners in eight Indonesian cities, Mahendradhata *et al.* (2015) found that only 24.6-74.3% of the GPs were familiar with the International Standards for Tuberculosis Care (ISTC), and 12.0-45.5% reported having prescribed second-line anti-tuberculosis drugs for newly diagnosed cases. ^13^ In an earlier survey of 174 private practitioners in Jogjakarta, Indonesia, Mahendradhata *et al.* (2007) found more than half (63.6%) had referred some patients suspected to have pulmonary TB to community health centres for diagnosis. ^14^ However, PPs still contribute a very small number of reported TB cases. Reasons for lack of reporting include lack of awareness about the mandatory nature of reporting and perceived reporting-associated work burden. ^15^ Therefore, the educational materials will cover both the NTP guidelines (published in 2016) and the most current international recommendations. The educational material will cover not only clinical management of TB patients but also public health and regulatory aspects of TB, from TB identification, to provisional diagnosis and referral, how to use the mobile phone app, and strategies to improve communication and increase adherence. Educational materials will be compiled in consultation with local experts. From the feasibility study, ^16^ we found that not all PPs have time to attend a 1-day training session; therefore, the education will be delivered as two 1-hour in-person sessions each separated 1-week apart ( Table 1). A training-for-trainers session will be held for each of the topic. Subsequently, we will do a follow-up visit, one month after the completed education, for every PP, followed by a 2-monthly electronic reminder. In addition, important aspects of daily TB management will be made available as desk references.

**Table 1.** Education topics and time allocation for each topic during the in-person education sessions.

Topic	Time allocation (minutes)
First educational visit	
Clinical aspects of TB	5
TB diagnosis	10
Principles of TB treatment	10
Diagnostic and therapeutic pathways for TB	10
Discussion	15
Second educational visit	
TB diagnosis referral pathways	10
TB treatment referral pathways	10
Recording and reporting aspects of TB	5
Using study-developed app for reporting TB cases	10
Discussion	15

**Figure 2.**
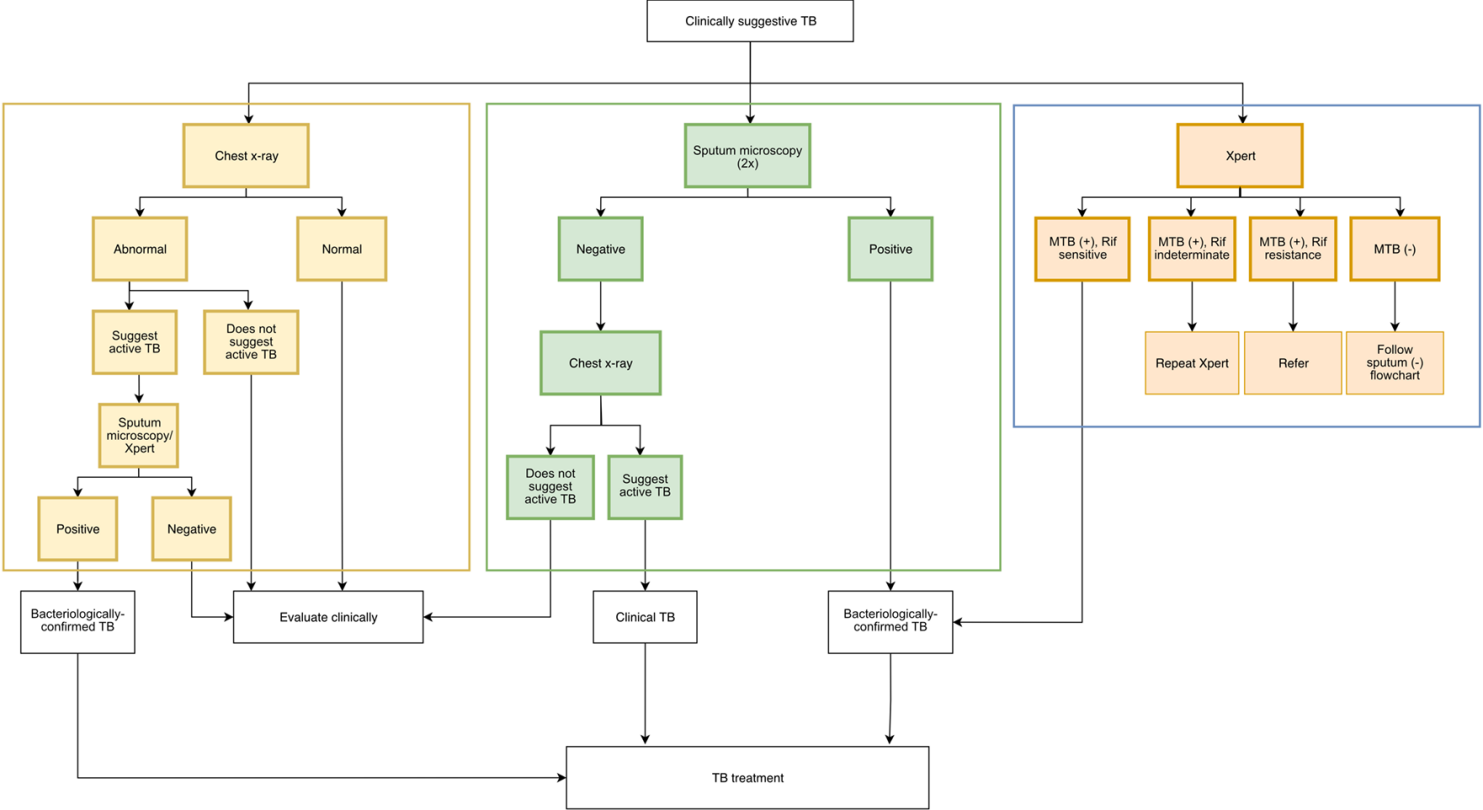
Pathways for diagnosis of TB to be selected by participating private practitioners (PPs). Sputum Xpert examination will be reserved for patients suspected for having drug-resistant TB, as per the local National Tuberculosis Programme guideline.

**Intervention 2: Patient management pathways.** Data from a previous study ^11^ will inform standardized TB management pathways for each PP, which take into account the context around each PP’s practice, identifying the most efficient and feasible diagnostic approach and notifying TB patients. ^17^ In consultation with clinical experts and NTP officers, information from WHO guidelines for radiography, ISTC, and the NTP, we will offer three possible pathways for diagnosing TB, all ensuring that bacteriological confirmation will be attempted for all patients. The first pathway starts with a chest radiograph, while the second starts with sputum smear microscopy, and the last, reserved for patients with suspected drug-resistant TB, starts with a sputum Xpert test ( Figure 2). Based on the NTP guidelines, Xpert use is currently limited for patients with presumptive drug-resistant TB. ^18^ PPs will be asked for their preferred pathway, although they may choose any pathway for any particular patient.

**Figure 3.**
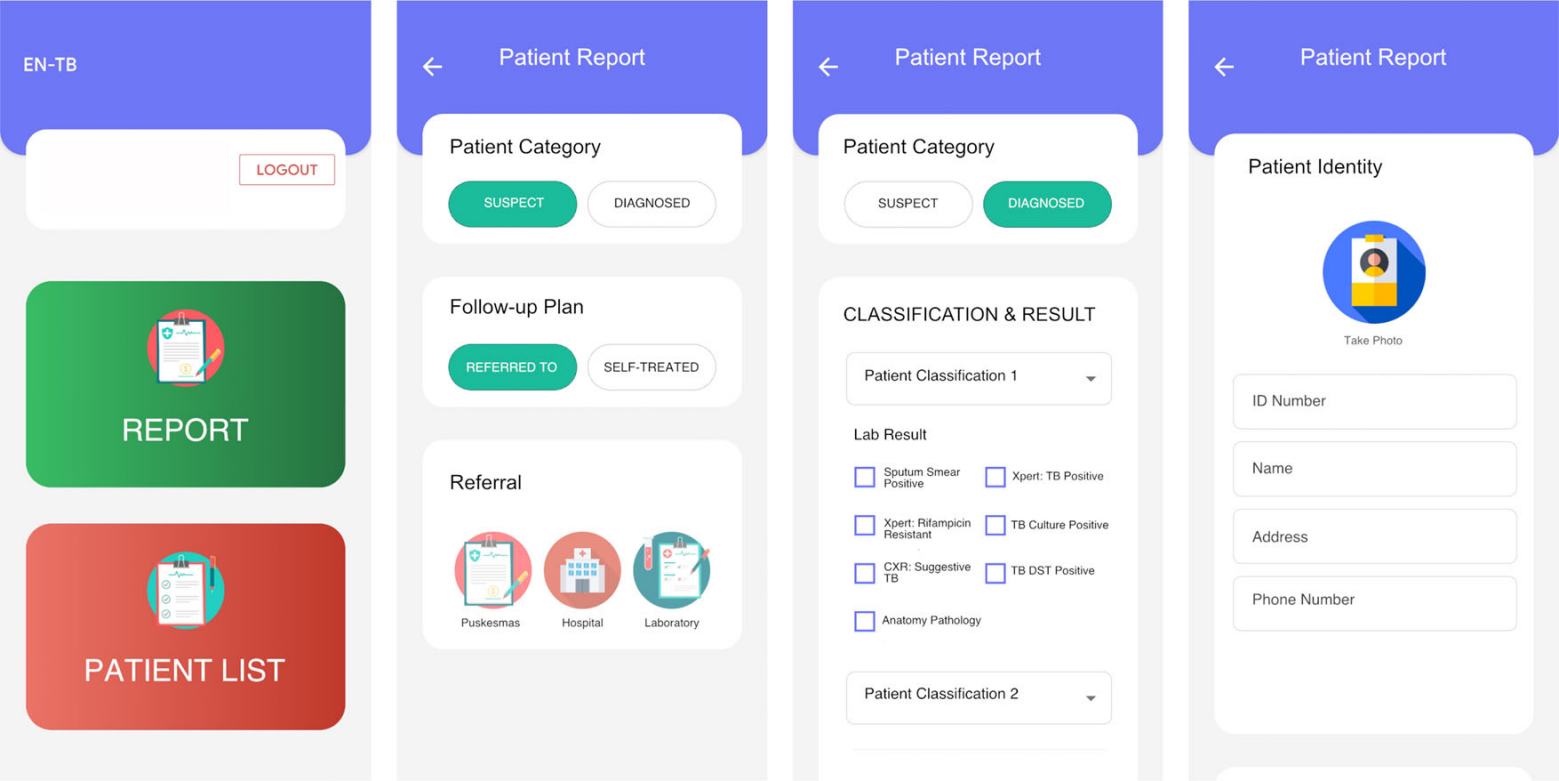
Illustration of the smartphone-based app that will be used to allow private practitioners to report essential information on suspected and diagnosed TB cases.

**Figure 4.**
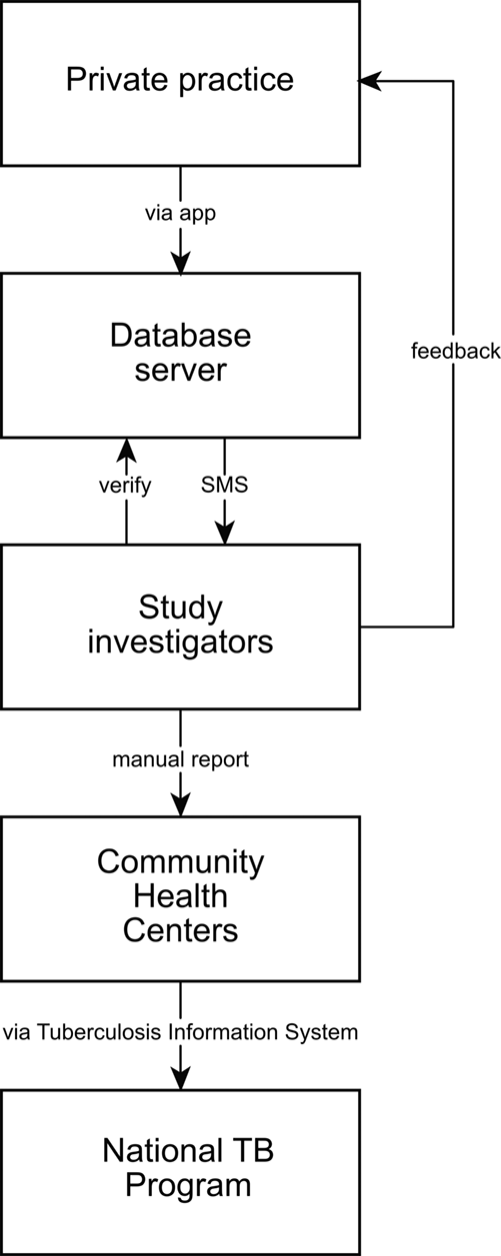
Flow chart of the electronic reporting system to notify the study team upon report of a new case.

**Intervention 3: Electronic system.** This system is a refinement of the previously developed electronic referral system using a mobile phone app compatible with both android and apple operating systems ( Figure 3). ^16^ The system will enable essential data to be uploaded, consistent with the NTP forms, which includes patient identity, diagnostic examinations, TB type, and referral plan. These variables will be selected in consultation with the NTP, taking into account PPs’ preferences that will be elicited by means of focus group discussions. Participating PPs will be asked to install and use the app for reporting, while keeping the patients informed. Participating PPs will inform the patients that the clinic is participating in a study about a TB notification system, and that patient information will be relayed to the Ministry of Health as per the regulatory requirement. Inputted information will be sent to a secure centralised server and will be monitored by authorized individuals in a web-based application. The server will generate an automated text message every time participating physicians add a record to the database. System development and piloting will include consultations with the NTP. Study investigators will manually report patient information to the NTP *via* CHC, as the electronic system is not currently linked to the NTP. If necessary, the study staff can contact the PPs or CHC officers to complete patient follow-up ( Figure 4).

### Criteria for discontinuing or modifying allocated interventions

Participating PPs are free to withdraw from the intervention at any time upon request. On the other hand, an investigator may discontinue or withdraw a PP from the intervention if the PP is unable to receive the study intervention within one month or if the PP meets an exclusion criterion that precludes further study participation. The reason for PP discontinuation or withdrawal from the study will be recorded.

### Strategy to improve adherence to intervention protocols

A follow-up will be made one month following the second educational visit. Due to the COVID-19 pandemic, the follow-up can be either an in-person visit or online meeting. This will facilitate additional discussion and to ensure that the PPs do not have any difficulties in understanding the recommended diagnostic pathways and using the reporting app. At the one-month visit, adherence to the intervention will be assessed according to pre-determined indicators, and a standard form filled with the results. At the end of the visit, any issues will be rectified through re-education. The intervention period will then be complete, while electronic refreshers will continue through fortnightly electronic messages. Once the PPs have received the intervention, real-time monitoring of referral practice of patients for diagnosis and notification will be undertaken through a secure web-link to the app.

## Outcomes

The primary endpoint is notifications of TB and will be measured in the 12 months before and the 12 months after the intervention is fully implemented. The change in the number of notifications will be compared between intervention clusters (n = 15) and control clusters (n = 15).

## Participant timeline

**Figure 5.**
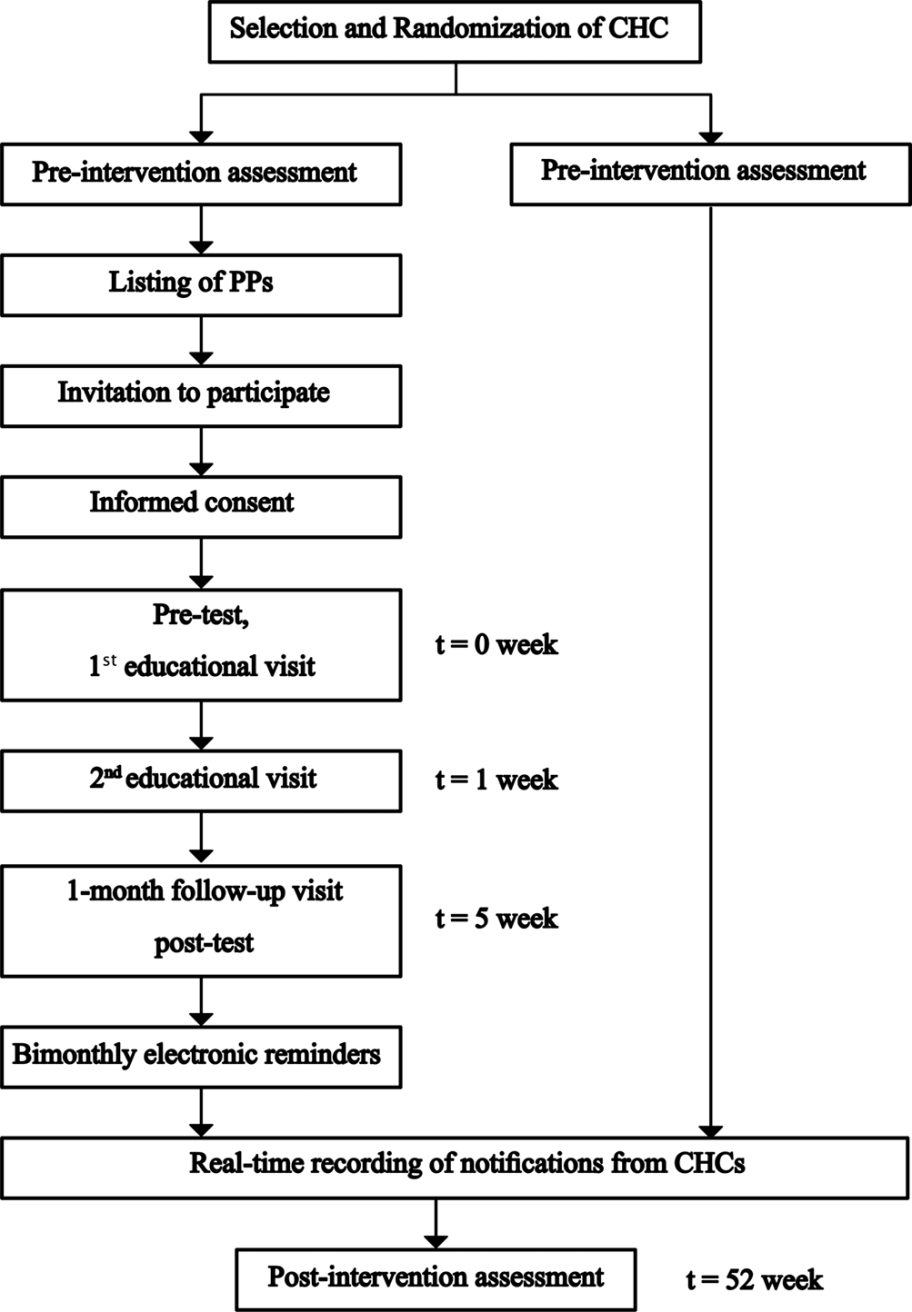
Timeline of selection, randomization and intervention of participating PPs.

The follow-up for participating PPs will be conducted as explained in Figure 5.

## Sample size

The primary analysis will compare the change in total number of notifications of patients between intervention and control areas. Less than 10% of PPs work privately in more than one CHC area. Those who also practice within the public system should not affect notifications from that public system, as it has a high notification rate (>85%) already. While the majority of notified TB patients are diagnosed and treated in their own CHC area, a number are diagnosed outside of their CHC area (up to 35%; data from our previous study). Those from a different CHC area will have an approximately one in five chance of being from another intervention area, a one in five chance of being from a control area, and a three in five chance of being from a non-study CHC area (n = 43 non-study CHC areas within Bandung). Therefore “contamination” of the intervention into control areas is estimated to be <10% (35% × 1/5 = 7%).

Assuming 1) PPs in each arm diagnose at least 500 TB patients in 12 months; 2) 65% of PP TB diagnoses are patients from their CHC area and 35% are from outside their area; and 3) a 15% baseline notification rate changing to 50% post-intervention in the intervention arm: PP notifications in the control arm areas will change from 75 to 87 notified cases while the change will be from 75 to 201 cases in the intervention arm. Total notifications (adding 475 per arm from non-PP) will change from 550 to 562 and 550 to 676, respectively. Taking the above into account, we will have approximately 90% power to detect a rate ratio of 1.2 (676/562) at the p = 0.025 level (one-sided).

## Recruitment

In the intervention arm, all eligible PPs who are identified through an updated list will be invited to participate in the study. For those whose contact numbers are obtained, research assistants will send up to three text/electronic messages to schedule a phone call to offer participation in the study; any PP who cannot be contacted by phone or does not respond to all three messages will be approached at their clinic. PPs whose contact numbers are not available will be directly visited by a research assistant. On the scheduled phone call/visit, a research assistant will explain about the study and offer participation after ascertaining that the PP meets all of the eligibility criteria. Upon agreement to participate, the research assistant will schedule a visit to obtain written informed consent and deliver the first education session.

## Assignment of interventions

### Allocation to intervention

We will randomly allocate 15 CHC areas as intervention areas, repeating the randomisation 100 times. From this we will select the 10 allocations that meet the criteria of 1) including having the least number of adjacent CHC areas between intervention and control arms, to minimize contamination between the arms, and 2) balancing by CHC numbers of TB cases diagnosed per annum. Then we will randomly select the final allocation from these ten.

### Blinding

Investigators, participating PPs and CHC staff will not be blinded to study arm as this is not practical in this type of trial. Staff conducting diagnostic investigations (laboratory personnel and radiologists) will not be part of the study team and will be blinded to study arm. Notification data will be abstracted from the local NTP registry so the data will not be influenced by study staff. Treatment allocation will be masked during data analysis. Trial randomisation codes will not be broken until the study is closed and analysis is commenced. Since there are no anticipated serious adverse events, no criteria for breaking the codes have been set.

## Data collection

From PPs in the intervention arm, prior to the intervention, we will collect information on their name, gender, age, and self-reported number of TB cases diagnosed in the last 3 months. This will enable comparison of basic characteristics between those who participate in the intervention and those who do not. PPs who refer patients for confirmation of diagnosis and notification will enter the following data about the patient into the app: name, gender, age, basis of presumptive/definitive diagnosis, other practice addresses, and the referral plan for the patient. The following data will be abstracted from routine records of TB cases at the CHCs: referring facility/practitioner, date, gender, patient identification, patient address (to the level of CHC area), sputum status, and basis of TB diagnosis.

Data abstraction from routine notification data will be done by trained enumerators. The 12-month period before intervention commences will be defined clearly and data abstraction will take place as soon as possible at the end of this period. The 12-month follow up period will be defined clearly, starting immediately after the last PP to receive the intervention has had their one-month follow up visit. Data abstraction will be done as soon as possible after this 12-month period is completed.

## Data management

Inputted information from PPs will be sent to a secure centralised server and will be monitored through log-in by authorized trial staff in a secure web-based application. The coordinating investigator is responsible for ensuring the accuracy, completeness, legibility, and timeliness of the data reported. Hardcopies of study visit worksheets will be provided for use as source document worksheets for recording data for each PP enrolled in the study. Data recorded in the electronic case report form derived from source documents will be consistent with the data recorded on the source documents.

Clinical and laboratory data, will be entered into a password-protected REDCap electronic database ^19^ and checked automatically for data that appear inconsistent, incomplete, or inaccurate. Clinical data will be entered directly from the source documents. Study documents will be retained for a minimum of 10 years after completion of the trial. No records will be destroyed without the written consent of the sponsor, if applicable. It is the responsibility of the sponsor to inform the investigator when these documents no longer need to be retained.

## Statistical analysis

### Primary analysis

The primary analysis will be intention-to-treat, where all patients notified by intervention and control PPs to a CHC will be included. Analysis will be carried out at the cluster level. 95% confidence intervals will be calculated and a significance level of 0.025 (one-sided) will be used. Study arms will be compared on baseline characteristics, including demographics of the PPs and the diagnosed TB cases, using descriptive statistics. We will estimate the rate ratio comparing TB notifications in the intervention and control groups. A generalised mixed model will be used with repeated measures of TB notification (pre- and post-intervention) and treatment×time interaction, a log link, Poisson errors and a random effect for PKM to account for over-dispersion.

### Secondary analysis

For the secondary objectives, the proportions of bacteriologically confirmed TB cases among the reported TB cases by PPs will be compared in intervention and control arms using a generalised estimation equation. The model will be fitted to repeated measures of the proportions of true positive TB notifications for each CHC (pre- and post-intervention) with a treatment by time interaction, a log link, and Poisson errors, with robust standard errors to allow for both the binary data and over-dispersion. Additionally, the primary trial analysis will be repeated excluding patients who were notified by a PP from a different CHC to their area of residence. Safety and interim analyses are not planned for the study.

## Safety

Safety oversight by a Data and Safety Monitoring Board (DSMB) will not be required for this public health intervention trial. However, an internal Data Monitoring Committee (DMC) will be established to oversee the study, focused on data quality. A quality management plan will be developed to monitor a site’s quality management. Quality control (QC) procedures will be implemented beginning with the data entry system and data QC checks that will be run on the database will be automatically generated on a weekly basis and any quality issues identified will be reviewed by the DMC and a plan put in place for resolution.

A series of standard operating procedures (SOPs) will be written to guide the research team and ensure the trial is conducted and data are generated, documented, and reported in compliance with the protocol.

## Ethics and dissemination

### Research ethics approval

Protocol and the consent forms have been reviewed and approved by the University of Otago human ethics committee (#H19-052), translated into Bahasa Indonesia and reviewed and approved by Universitas Padjadjaran ethics committee (#1089/UN6.KEP/EC/2019).

### Protocol amendments

Any major modifications to the protocol including changes of study objectives, study design, sample sizes, study procedures, or significant administrative aspects will be submitted for approval by the University of Otago and Universitas Padjadjaran’s ethics committees.

### Consent

Consent forms describing the study are given to the head of each CHC institution. For PPs undergoing intervention, consent forms describing in detail the study intervention, study procedures, and risks are given to the PP. The investigator will explain the research study to the participant PP and answer any questions that may arise. A verbal explanation will be provided in terms suited to the PP’s comprehension of the purposes, procedures, and potential risks of the study and of their rights as research PPs. PPs will have the opportunity to discuss the study with their family or surrogates, carefully review the written consent form and ask questions prior to signing. PPs will be informed that participation is voluntary and that they may withdraw from the study at any time, without prejudice. The consent process will be conducted the form signed prior to any intervention and a copy of the document given to PPs for their records. The rights and welfare of the PPs will be protected by emphasizing to them that there will be no negative repercussions if they decline to participate in this study, including no reporting of discontinuation to government authorities.

### Confidentiality

Participating PPs’ confidentiality and privacy are extended to cover any information relating to them. Therefore, the study protocol, documentation, data, and all other information generated will be held in strict confidence. No information concerning the study or the data will be released to any unauthorized third party without prior written approval of the sponsor. All research activities will be conducted in a setting that is as private as possible.

PP research data, which are for purposes of statistical analysis and scientific reporting, will be stored. This will not include the PP’s contact or identifying information. Rather, individual PPs and their research data will be identified by a unique study identification number. The study data entry and study management systems will be secured and password protected. At the end of the study, all study databases will be de-identified and archived.

### Declaration of interests

Any actual conflict of interest of persons who have a role in the design, conduct, analysis, publication, or any aspect of this trial will be disclosed and managed. Furthermore, persons who have a perceived conflict of interest will be required to have such conflicts managed in a way that is appropriate to their participation in the design and conduct of this trial. The study leadership has established policies and procedures for all study group members to disclose all conflicts of interest and will establish a mechanism for the management of all reported dualities of interest.

### Access to data

Paper-based study data will be stored in a locked cabinet in the Tuberculosis Working Group office in Bandung. All electronic and web-based data will be secured by appropriate security protocols, password protected and only accessible to study investigators and designated research assistants.

Data collected for this study will be analysed and stored. After the study is completed, the de-identified, archived data may be transmitted to and stored, for use by other researchers including those outside of the study. Permission to transmit data will be included in the informed consent.

### Dissemination policy

This study is not subject to any particular publication and data-sharing policies and regulations. However, we aim to publish study results in scientific conferences and a peer-reviewed journal, as well as presenting the results to relevant stakeholders in TB control in Indonesia.

## Discussion

To our knowledge, the use of a randomized controlled trial to evaluate the effect of intervention to increase PPs involvement in TB control has been reported just once in the past ten years. ^20^ Yellapa *et al*. implemented a package of interventions to PPs in the city of Tumkur, South India, and reported an almost a two-fold increase in case referral from intervention-arm PPs. ^20^ Our study has a larger planned sample size and more focus on increasing notifications which was not sufficiently impacted in the aforementioned study. Additionally, using cluster-randomization and taking into account clustering in the analysis is more appropriate to measure the effect of programmatic intervention where implementation is being done at the cluster level. Hence, our study will contribute to providing robust evidence about the effect of intervening PPs on increasing TB patient notification and referral, in particular in the context of an urban setting in a lower middle-income country.

The study intervention is given as a package; therefore, it will be difficult, if not impossible, to separate out any individual intervention’s effect on TB notification and referral. However, pre- and post-test data may be used to measure the impact of in-person education on PPs’ knowledge and, insofar as knowledge correlates with practice, evidence of an increase in knowledge may suggest that education can potentially improve practice. However, it is understood that knowledge does not directly translate into practice and a “know–do” gap has been observed. ^21^

For practical reasons, it will not be possible to blind the study investigators and research assistants to treatment allocation. To reduce potential bias due to differential rigor in data collection and analysis, the allocation will be concealed from enumerators responsible for collecting the study outcome data as well as in the data set used in analyses.

The study area is relatively small and homogeneous, we also limit participation to general practitioners and a limited number of specialties (internists and pulmonologists) who are the most likely PPs to refer TB cases. ^20^ This study nonetheless will provide evidence of effectiveness for the package of interventions aimed at PPs and will be generalizable to similar settings in Indonesia, which, in itself, is important in the context of TB control.

As mentioned, a small amount of contamination between intervention and control arms is likely. PPs can practice in more than one place that may be located in both of the study arms. Further, PPs may also practice in the public sector, including at CHC. Lastly, it will not be possible to conceal the study intervention from TB programme officers in the control arm because these officers are in regular communication with one another. We assume that the degree of contamination will be small.

Cluster-randomised trials can be subject to selection bias if participants are recruited after randomisation, as is being done in this study. ^22^ However, selection bias can be minimized because inclusion and exclusion criteria have been set a priori and participation will be offered to every PP meeting those criteria identified from mapping conducted before the start of the study. Lack of adherence of PPs in completing interventions was found to be a common problem in similar studies. ^23^ Although participation is voluntary and hence PPs do not have to provide reasoning for withdrawing from the study, we will record reasons if they are provided and this information can be used to inform strategies to increase adherence.

Increasing PPs involvement in TB-PPM requires a PP-centred approach. This study will provide valuable evidence on whether a PP-centred intervention package is effective in increasing the number of TB notifications and referrals. In addition, health care facilities/providers are required to report the time of diagnosis, the time of treatment initiation, and the means by which TB is diagnosed. The potential effect of the intervention to these outcomes may be measurable from routine reporting data that will be collected, although we will not be able to measure the delay to treatment initiation in the control arm because we are not collecting data from PPs in that arm. The study results will be important to guide policy in TB-PPM in Indonesia and other similar settings.

## Data availability

### Underlying data

No data are associated with this article

### Extended data

Open Science Framework: Extended data for ‘Protocol for increasing notifications of tuberculosis from private practitioners (INSTEP2): A randomised controlled trial’, https://doi.org/10.17605/OSF.IO/KAGNB. ^24^

Data are available under the terms of the Creative Commons Zero “No rights reserved” data waiver (CC0 1.0 Public domain dedication).

### Reporting guideline

Open Science Framework: SPIRIT 2013 checklist for ‘Protocol for increasing notifications of tuberculosis from private practitioners (INSTEP2): A randomised controlled trial’, https://doi.org/10.17605/OSF.IO/KAGNB. ^24^

Data are available under the terms of the Creative Commons Zero “No rights reserved” data waiver (CC0 1.0 Public domain dedication).

### Author roles

**PFH:** Data Curation, Formal Analysis, Investigation, Methodology, Project Administration, Resources, Writing – Original Draft Preparation, Writing – Review & Editing. **BWL:** Data Curation, Formal Analysis, Investigation, Methodology, Project Administration, Resources, Writing – Original Draft Preparation, Writing – Review & Editing. **KS:** Methodology, Formal Analysis, Supervision, Writing – Review & Editing. **NA:** Data Curation, Investigation, Project Administration, Writing – Review & Editing. **LC:** Data Curation, Writing – Review & Editing. **CCH:** Methodology, Formal Analysis, Writing – Review & Editing. **SM:** Supervision, Writing – Review & Editing. **RvC:** Methodology, Writing – Review & Editing. **MM:** Funding Acquisition, Methodology, Resources, Supervision, Writing – Review & Editing. **BA:** Conceptualization, Data Curation, Funding Acquisition, Investigation, Methodology, Resources, Supervision, Writing – Review & Editing. **PH:** Conceptualization, Funding Acquisition, Methodology, Resources, Supervision, Writing-Original Draft Preparation, Writing – Review & Editing.
