# Supplementary material for: A public health intervention package for increasing tuberculosis notifications from private practitioners in Bandung, Indonesia (INSTEP2): A cluster-randomised controlled trial protocol
Source: F1000Res. 2022 Mar 25;10:327. Originally published 2021 Apr 27. [Version 2] doi: 10.12688/f1000research.52089.2 (PMC9039369; doi:10.12688/f1000research.52089.2)
Supplement: Peer review history

# Peer review: Reviewer response for version 2

**DOI:** 10.5256/f1000research.122604.r128809

**Published:** 2022-04-25

**Authors:** Saurav Basu

**Affiliations:** 1 Public Health Foundation of India, Gurgaon, India

Based on the changes, I approve the trial protocol for indexing.

Is the study design appropriate for the research question?

Yes

Is the rationale for, and objectives of, the study clearly described?

Yes

Are sufficient details of the methods provided to allow replication by others?

Yes

Are the datasets clearly presented in a useable and accessible format?

Not applicable

Reviewer Expertise:

Public health, Epidemiology and Community Medicine

I confirm that I have read this submission and believe that I have an appropriate level of expertise to confirm that it is of an acceptable scientific standard.

# Peer review: Reviewer response for version 1

**DOI:** 10.5256/f1000research.55317.r95006

**Published:** 2021-10-27

**Authors:** Charity Omenka

**Affiliations:** 1 Department of Epidemiology, Biostatistics and Occupational Health, McGill University, Montréal, QC, Canada

This protocol describes a cluster-randomised control trial involving deployment of a multi-component intervention package. The methods, including the setting and eligibility criteria are clearly defined.

 However, the authors need to clarify the 5 components of the intervention as only 3 interventions are defined.

 It would also be useful if the authors compared the primary outcome - change in notifications - between the 3 diagnostic methods mentioned - chest radiograph, sputum microscopy and Xpert testing.

Is the study design appropriate for the research question?

Yes

Is the rationale for, and objectives of, the study clearly described?

Yes

Are sufficient details of the methods provided to allow replication by others?

Yes

Are the datasets clearly presented in a useable and accessible format?

Not applicable

Reviewer Expertise:

Access to TB services

I confirm that I have read this submission and believe that I have an appropriate level of expertise to confirm that it is of an acceptable scientific standard.

# Response

**Published:** 2022-03-18

**Authors:** Bony Wiem Lestari

**Affiliations:** Department of Public Health, Faculty of Medicine Universitas Padjadjaran, Bandung 40161, Indonesia , Indonesia

Thank you for your valuable comments. Please find below our specific responses:

**Comment-1: **However, the authors need to clarify the 5 components of the intervention as only 3 interventions are defined.

**Response:**

 We apologise for the inconsistency. The five components of intervention are: 1) electronic referral and notification system, 2) standardized education materials, 3) an individualised plan for each PP with respect to their approach to the diagnosis and management of TB suspects, 4) 1 month follow-up visit from the study team, and 5) 2-monthly electronic reminder/refresher. However, components 4 and 5 are included as parts of the standardized education component. We have revised the statement in our manuscript to state that the intervention consists of three components.

**Comment-2:** It would also be useful if the authors compared the primary outcome - change in notifications - between the 3 diagnostic methods mentioned - chest radiograph, sputum microscopy and Xpert testing.

**Response:**

 Thank you for your suggestions. Indeed, health care facilities/providers are required to report the time of diagnosis, the time of treatment initiation, and the means by which TB is diagnosed (e.g., via sputum microscopy vs clinically). The potential effect of the intervention to these outcomes may be measurable, although we will not be able to measure the delay to treatment initiation in the control arm because we are not collecting data from PPs in this arm. We have added this in the discussion section.

# Peer review: Reviewer response for version 1

**DOI:** 10.5256/f1000research.55317.r90373

**Published:** 2021-08-12

**Authors:** Saurav Basu

**Affiliations:** 1 Public Health Foundation of India, Gurgaon, India

This study protocol describes the implementation of a public health intervention package through a cluster RCT to improve TB notification rate and awareness of PPs who are involved in the management of patients with TB. 

 The intervention comprises of three components including educational, patient management pathways, and an electronic system. 

 The protocol needs to provide more detail on the development of the intervention package, especially in context of the educative module (what is the baseline knowledge of TB in the PPs?). Reasons for non-notification also need to be explained and how this intervention package will promote notification (reasons other than lack of knowledge can also exist).

 You can include secondary outcomes such as delay in treatment initiation (time from presentation to PP and initiation of ATT).

Is the study design appropriate for the research question?

Yes

Is the rationale for, and objectives of, the study clearly described?

Yes

Are sufficient details of the methods provided to allow replication by others?

Yes

Are the datasets clearly presented in a useable and accessible format?

Not applicable

Reviewer Expertise:

Public health, Epidemiology and Community Medicine

I confirm that I have read this submission and believe that I have an appropriate level of expertise to confirm that it is of an acceptable scientific standard, however I have significant reservations, as outlined above.

# Response

**Published:** 2022-03-18

**Authors:** Bony Wiem Lestari

**Affiliations:** Department of Public Health, Faculty of Medicine Universitas Padjadjaran, Bandung 40161, Indonesia , Indonesia

We thank the reviewer for their valuable comments. Please find below our specific responses.

**Comment-1: **The protocol needs to provide more detail on the development of the intervention package, especially in context of the educative module (what is the baseline knowledge of TB in the PPs?). Reasons for non-notification also need to be explained and how this intervention package will promote notification (reasons other than lack of knowledge can also exist).

**Response:**

 We have revised the manuscript to include a more detailed information about the development of the intervention package, especially the education component.

**Comment-2: **You can include secondary outcomes such as delay in treatment initiation (time from presentation to PP and initiation of ATT).

**Response:**

 Indeed, health care facilities/providers are required to report the time of diagnosis, the time of treatment initiation, and the means by which TB is diagnosed. The potential effect of the intervention to these outcomes may be measurable, although we will not be able to measure the delay to treatment initiation in the control arm because we are not collecting data from PPs in this arm. We have added this in the discussion section.
